# Aldosterone and Mineralocorticoid Receptor System in Cardiovascular Physiology and Pathophysiology

**DOI:** 10.1155/2018/1204598

**Published:** 2018-09-19

**Authors:** Alessandro Cannavo, Leonardo Bencivenga, Daniela Liccardo, Andrea Elia, Federica Marzano, Giuseppina Gambino, Maria Loreta D'Amico, Claudia Perna, Nicola Ferrara, Giuseppe Rengo, Nazareno Paolocci

**Affiliations:** ^1^Center for Translational Medicine, Lewis Katz School of Medicine, Temple University, Philadelphia, USA; ^2^Department of Translational Medical Science, Federico II University of Naples, Naples, Italy; ^3^Istituti Clinici Scientifici Maugeri SpA, Telese Terme, Italy; ^4^Division of Cardiology, Johns Hopkins University, Baltimore, MD, USA

## Abstract

The mineralocorticoid hormone aldosterone (Aldo) has been intensively studied for its ability to influence both the physiology and pathophysiology of the cardiovascular system. Indeed, although research on Aldo actions for decades has mainly focused on its effects in the kidney, several lines of evidence have now demonstrated that this hormone exerts disparate extrarenal adverse effects, especially in the circulatory system. Accordingly, in the last lusters, a number of studies in preclinical models (*in vitro* and *in vivo*) and in humans have established that Aldo, following the interaction with its receptor—the mineralocorticoid receptor (MR)—is able to activate specific intracellular genomic and nongenomic pathways, thus regulating the homeostasis of the cardiovascular system. Importantly, through this mechanism of action, this hormone becomes a crucial regulator of the function and growth of different types of cells, including fibroblasts, cardiomyocytes, and vascular cells. For this main reason, it is plausible that when Aldo is present at high levels in the blood, it profoundly modifies the physiology of these cells, therefore being at the foundation of several cardiovascular disorders, such as heart failure (HF). On these grounds, in this review, we will provide an updated account on the current knowledge concerning Aldo activity in the cardiovascular system and the most recent preclinical studies and clinical trials designed to test better approaches able to counter the hyperactivity of the Aldo/MR signaling pathway in the setting of cardiovascular diseases.

## 1. Introduction

The mineralocorticoid aldosterone (Aldo) is a steroid hormone synthesized and secreted in response to renin-angiotensin system activation (RAS) or high dietary potassium by the zona glomerulosa (ZG) of the adrenal cortex [1]. At cellular level, Aldo activity is dependent by the binding and activation of the cytoplasmic/nuclear mineralocorticoid receptor (MR) [2]. To this respect, in distal renal tubular cells, the Aldo/MR system mainly controls renal sodium retention and potassium excretion augmenting intravascular volume [3]. Currently, an increasing body of evidence suggests that alteration in Aldo levels and the consequent MR hyperactivation can be either responsible or a factor in the onset and progression of several cardiovascular disorders, including metabolic syndrome, hypertension, coronary artery disease (CAD), chronic kidney disease, and congestive heart failure (CHF) [4]. For instance, in cardiomyocytes and in vascular cells (vascular smooth muscle cells (VSMC) and endothelial cells), Aldo downstream MR negatively affects function, growth, and survival [5, 6]. In contrast, in fibroblasts Aldo acts as an enhancer of their function, increasing both proliferation and migration [7–9]. All these deleterious effects have been found to be associated to the onset and development of cardiac disease, including HF [10]. For this reason, there is a keen interest in the identification of specific therapies able to block the hyperactivity of this noxious hormone and its receptor [10]. To this end, a number of preclinical and clinical studies have been designed in order to test the effects of these treatments. Hence, in this review we will guide the reader towards the current knowledge of Aldo/MR system function in the cardiovascular system, while providing an up-to-date overview about all the pharmacological, and not, strategies directed to abolish its deleterious effects, directly via inhibition of MR or indirectly via inhibition of the renin-angiotensin system (RAS).

## 2. Aldosterone: From Biosynthesis to Release

Synthesis and release of Aldo, by the adrenal cortex, are promoted by the RAS and potassium ions (K^+^) that are considered main triggering factors, as well as by minor stimulators like adrenocorticotropin (ACTH) and vasopressin (VP) [11–15]. Conversely, other molecules such as dopamine and atrial natriuretic peptide (ANP) exert an inhibitory effect on Aldo production/release [14, 16].

### 2.1. RAS

The effect of sodium is undoubtedly mediated by increased RAS activity [17]. Indeed, renin, a protease synthesized by the juxtaglomerular apparatus of the kidney, is secreted in response to reduced renal blood flow, hemorrhage, dehydration, and salt restriction. Renin induces the cleavage of angiotensinogen to form angiotensin I (Ang I) which in turn is converted by the Ang-converting enzyme (ACE), to the active peptide Ang II [17]. Finally, Ang II binding to the Ang II type 1 receptor (AT1R) promotes the delivery of cholesterol to the inner mitochondrial membrane and of the activation of aldosterone synthase, via enhancement of CYP11B2 gene transcription. Aldo synthase, which exhibits a high 11*β*-hydroxylase, then completes the synthesis of the hormone [17].

### 2.2. Potassium

The mechanism of action of K^+^ is independent of RAS. In particular, these ions depolarize the plasma membrane and activate voltage-dependent calcium (Ca^2+^) channels, thus allowing the influx or efflux of extracellular Ca^2+^ [18]. This Ca^2+^ current induces the activation of calmodulin- (CAM-) dependent kinase, which activates in turn transcription factors and members of the CRE-binding protein family, thus promoting CYP11B2 gene expression with consequent increase in Aldo biosynthesis and release [15, 19].

### 2.3. ACTH

The hormone ACTH represents the primary regulator of cortisol production. Importantly, following the interaction with glomerulosa cell-surface melanocortin-2 receptor (MC2R), a G protein-coupled receptor (GPCR), ACTH, activates the adenylate cyclase that in turn promotes the formation of cyclic adenosine monophosphate (cAMP), thus enabling the transfer of cholesterol to the inner membrane of the mitochondria and the synthesis of adrenal steroids, such as Aldo [18].

### 2.4. VP

Also known as antidiuretic hormone (ADH), VP has a crucial role in osmoregulation because it controls the amount of urine formation. Importantly, several studies have demonstrated that, although modestly, VP can exert stimulatory effects on Aldo synthesis from ZG cells [20]. Importantly, this effect appears to be dependent on the activation of its VP receptor with a subsequent activation of Aldo synthase [20].

### 2.5. Dopamine

Dopamine represents the predominant neurotransmitter in the human central nervous system (CNS), controlling a variety of functions [21]. Importantly, dopamine is also a regulator of cardiovascular and renal function. Its functions are all mediated by the binding to dopamine receptors. Notably, in human normal adrenal glands, dopamine 2 and dopamine 4 receptors are expressed both in the adrenal cortex and in the medulla. In the cortex, these structures are localized mainly in the ZG and are liable for Aldo synthesis [21, 22]. Consistent with this, several reports have demonstrated that the administration of D2 antagonists, such as metoclopramide, increases Aldo concentrations in the plasma [21, 22].

### 2.6. ANP

ANP is released by the heart, in particular in the atria, in response to elevated atrial pressure [16]. This peptide (28 amino acids) is considered one of the most important inhibitors of Aldo synthesis [16]. The mechanism of action of ANP appears to be mediated, at least in part, by interfering with extracellular Ca^2+^ influx and by the inhibition of RAS, K^+^, and ACTH [16].

## 3. Structure and Function of Mineralocorticoid Receptor

Originally recognized as isoform 1 of the corticosteroid receptor because of its capability to bind with equal affinity to both Aldo and cortisol, the MR was identified in 1987 by Arriza and coworkers [23], as the unique Aldo receptor. The MR is a transcription factor that, following its binding to Aldo in the cytosol, translocates to the nucleus, leading to changes in gene expression in the target tissues [10, 24, 25].

**Figure 1:**
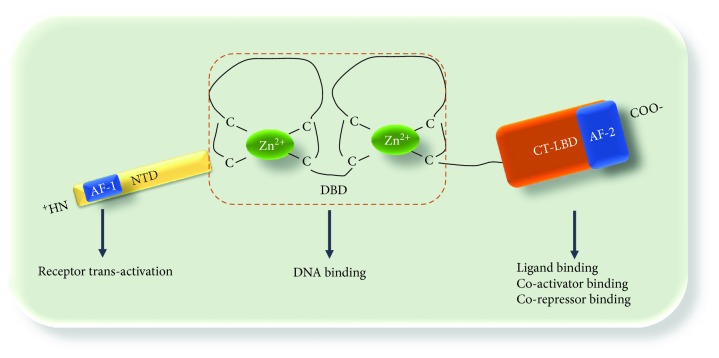
Schematic representation of MR structure: the MR is constituted of three domains: an amino-terminus domain (NTD), which contains a NH_2_-terminal ligand-independent transcriptional activation (AF-1) and contributes to transactivation of other receptors; a central cysteine-rich DNA-binding domain (DBD), which contains two zinc fingers (ZFs); and a carboxy-terminal- (CT-) ligand-binding domain (CT-LBD) which contains an activation function 2 domain (AF-2), which is crucial for ligand and coregulator binding.

Importantly, the MR has a structural affinity with the nuclear receptor superfamily (NR) constituted of three principal domains [26–29]: an amino-terminus domain (NTD), a central cysteine-rich DNA-binding domain (DBD), and a carboxy-terminal- (CT-) ligand-binding domain (CT-LBD) (Figure 1). The NTD (about 602 amino acids) cooperates synergistically with the CT-LBD to reinforce the receptor structure [28, 29]. The DBD (about 66 amino acids) is highly conserved within the NRs and contains two zinc fingers (ZFs), each composed by four cysteine residues connected tetrahedrally with zinc ion (Zn^2+^), by a disulfide bond [28, 29]. The DBD and the CT-LBD are connected by a linker domain. The CT-LBD is composed of 251 amino acids and consists of 12 *α*-helices orientated and four *β*-strands generating three antiparallel layers [30–32]. Interestingly, in the presence of a ligand, helix 12 forms a cavity, termed activation function 2 domain (AF-2). Inside this structure, coactivator molecules with a leucine-rich motif connect. The NTD is mostly unstructured and contributes to the transactivation of other receptors (see below), via AF-1 [28].

Interestingly, among MR coactivators identified so far, peroxisome proliferator-activated receptor gamma coactivator 1-alpha (PGC-1*α*) which is implicated in energy balance and metabolic modulation appears to interact with the AF-2 domain of MR [33]. Importantly, the physiological meaning of MR-mediated activation of PGC-1α is still unclear and under debate. In fact, recent reports have documented that, in renal tissues, this factor and MR are not coexpressed [34, 35]. However, the evidence that PGC-1α is expressed at high levels in brown adipose tissue, where MRs participate in adipocyte differentiation [36], suggests the existence of ligand and cell-specific MR coactivators [37, 38]. For instance, the enzyme, 11beta-hydroxysteroid dehydrogenase type II (HSD2), which prevents the binding of glucocorticoids to the MR by inactivating cortisol to the inactive metabolite 11-dehydrocorticosterone, confers to MR a high degree of selectivity for Aldo [39]. In contrast, in cells lacking this enzyme (endothelial cells and VSMCs), the MR interacts with both Aldo and cortisol with a similar affinity [40, 41].

Further to the effects mediated by coactivators, ligands such as glucocorticoids have been showed to block aldosterone effects, in both neuronal cells and cardiomyocytes [42–44]. Indeed, corticosterone has been shown to inhibit Aldo-induced hypertrophy in cardiomyocytes. Moreover, as demonstrated by Rossier and colleagues [45], chronotropic response in cardiomyocytes was activated only by Aldo but not by corticosterone. Hence, all these data concur to attest that all Aldo-mediated effects are mainly dependent on ligand-, gene-, and cell-selective MR answers as well as specific coactivators.

## 4. Signaling Pathways Activated Downstream Aldosterone/Mineralocorticoid Receptor System: Genomic and Nongenomic

As discussed above, thanks to its particular structure, the MR acts mainly as a transcription factor. For this very reason, for many years all Aldo effects have been confined to the regulation of gene expression via activation of the “genomic” pathway [46]. Importantly, this particular function of MR appears to be strictly dependent on CT-LBD and on specific binding with heat shock proteins (HSPs) [47]. Indeed, in its cytoplasmic inactive form, MR is bound to chaperones Hsp70 and 90 and various immunophilins [47, 48]. However, upon Aldo binding, these molecules dissociate from the CT-LBD, thus allowing the activation and subsequent nuclear translocation of the MR [49, 50]. In the nucleus, the MR binds to hormone response elements (HRE) within the promoter region of target genes [49], inducing either the activation or the repression of gene transcription. This effect is dependent on coactivators or corepressors that are recruited at the gene-transcription initiation complex [51].

Importantly, the regulation of gene expression occurs in an early phase (hours from Aldo stimulation) and in a later phase, when new pumps, ion channels, and transporters are directly produced and their density is augmented at the plasma membrane level. For instance, the epithelial sodium channel (ENaC) is considered a final effector of Aldo/MR system activation in the kidney. Especially in renal epithelial cells, ENaC promotes sodium and water reabsorption, regulating body fluid volume and blood pressure. [25]. Serum glucocorticoid-regulated kinase 1 (SGK1) is another important molecule acting downstream of the Aldo/MR system [52]. Interestingly, SGK1 is a serine-threonine kinase, activated early (≅30 minutes) after Aldo stimulation, and its activity has been related to the increased cell surface density of ENaC. In particular, SGK1 phosphorylates the ubiquitin ligase Nedd 4-2, inhibiting it and thus blocking the degradation of ENaC [52]. Of note, in response to Aldo stimulation, SGK1 has been demonstrated to enhance the expression of several profibrotic genes in the myocardium, such as the connective tissue growth factor (CTGF). This evidence lends further support to the notion that Aldo/MR activity has pathophysiologically relevant extrarenal influence [53]. In keeping with this, it has been shown that Aldo/MR system activation in cardiomyocytes exerts prohypertrophic effects, via the enhancement of fetal gene expression [53]. More in detail, Yoshida and coworkers [53] have recently reported that, by interacting with p300 and GATA4, the MR activates the ANP pathway, with a consequent increase in cardiomyocyte size.

Beyond the activation of such genomic pathways, Aldo is also able to rapidly activate specific molecular pathways and typically in a few minute time range. These “nongenomic phenomena” can be either MR-dependent or -independent (i.e., likely related to another receptor) or possibly mediated by a cross-talk mechanism [44, 54]. For instance, in renal epithelial cells, it has been shown that Aldo stimulation results in a rapid and transient increase in intracellular Ca^2+^ [55, 56]. Of note, as demonstrated by Grossmann and colleagues [57], this effect is dependent on the activation of the tyrosine kinase c-Src and the consequent transactivation of the epidermal growth factor receptor (EGFR). After the activation of the EGFR signaling pathway, it has been proposed also a role for the mitogen-activated protein kinase ERK, the protein kinase C (PKC) [56, 58], and PI3K [59]. In this context, it is worth stressing that c-Src is a key player in these nongenomic pathways. Indeed, upon Aldo stimulation of VSMCs with Aldo, c-Src activates p38 mitogen-activated protein (MAP) kinase and nicotinamide adenine dinucleotide phosphate oxidases (NOX) 2 and 4 with a subsequent increase in collagen synthesis and reactive oxygen species generation (ROS) [60, 61]. Interestingly, the activation of c-Src, as also ERK, downstream Aldo, appears to be either G protein-dependent or -independent. In this regard, we and others have demonstrated that ERK activation can be blocked through the inhibition of the AT1R, suggesting the presence of a cross-talk between Aldo and Ang II signaling pathways [54, 62]. Indeed, in the early 80s, it has been reported that, in VSMCs, MR and AT1R interact synergistically to modulate the function of these cells [63–65]. Similarly, other reports have documented that the activation of the MR/AT1R system is responsible for both the genomic and nongenomic effects associated to Aldo stimulation [63, 64]. Importantly, in cardiomyocytes, we have recently documented that following Aldo stimulation there is the activation of c-Src/beta arrestin endocytic machinery at the plasma membrane which leads to AT_1_R internalization, followed by the activation of ERK 1/2 and NOX4 [54]. Of note, this mechanism appears to be liable for mitochondrial ROS generation and increased apoptotic response observed in presence of high Aldo levels. Moreover, owing to MR-AT_1_R system activation, we have also described that Aldo induces the nuclear translocation of GPCR kinase 5 (GRK5). Interestingly, along this pathway, GRK5 appears to be a major player in the “genomic” pathway of Aldo-mediated hypertrophy, more specifically through the activation of myocyte enhancer factor 2 (MEF2) [54].

Further to EGFR and AT1R transactivation, others have documented that Aldo is able to activate the estrogen receptor GPER in both myocytes (H9c2 cells) and nonmyocytes (vascular smooth muscle cells (VSMCs)) [66–68]. However, according to a recent study by Ashton and colleagues [66], this signaling pathway may not be sufficient to induce deleterious effects in the myocardium and in VSMCs. In keeping with this evidence, we have observed that GPER activation, at least in neonatal cardiomyocytes, has a minor impact on Aldo signaling activation [54, 69].

## 5. Increased Aldosterone Levels and Consequent Adverse Effects in the Cardiovascular System

Since its isolation by Simpson et al. in the 1950s, all the Aldo-mediated effects were originally believed to be confined to just a few target organs of epithelial origins [69, 70]. However, in the last decades, a number of studies have expanded the spectrum of compartments and tissues in which Aldo appears to exert its activity [69]. Consistent with this new scenario, reports have shown that the (patho-)physiology of heart and blood vessels, for instance, is significantly affected by the action of this hormone [69]. Indeed, Aldo is a well-recognized inducer of cardiac fibrosis and hypertrophy, and it acts as an enhancer of inflammatory cell function, thus negatively impacting on vascular cell function and growth [71, 72]. Furthermore, disturbances in cardiac rhythm have been observed in the presence of elevated circulating levels of Aldo [72]. It is noteworthy that, in the long run, all these effects significantly contribute to the onset of cardiovascular disease and to its progression to HF.

Several histopathological reports have documented a direct toxic role of Aldo on the vasculature, mostly obtained in the context of rat models of hypertension [73–75]. In particular, as shown by some studies, Aldo is able to induce an adverse structural remodeling of the vasculature, with actions that are nearly abolished by MR antagonism [73–75]. In line with this evidence, Rocha and colleagues [76] reported that, in uninephrectomized rats, Aldo leads to the development of severe hypertension and vascular inflammation in the coronary arterioles. In particular, MR activation increases oxidative stress in the vessel walls and induce an inflammatory response. Indeed, Aldo stimulation results in the upregulation of several inflammatory factors such as MCP-1, CCL2, CX3CL1, and CCL5 and adhesion molecules including the intercellular adhesion molecule-1 (ICAM-1) and the vascular cell adhesion molecule-1 (VCAM-1) [77]. It is worth stressing that either Aldo-induced cardiac fibrosis or hypertrophy has been proposed as a mere consequence of the inflammatory events mediated by this hormone, when present at high levels. Specifically, as demonstrated by Rickard and colleagues [78] and by Bienvenu and coworkers [79], macrophages lacking MR are more protected from fibrotic and proinflammatory stimuli such as deoxycorticosterone acetate (DOCA)/sodium chloride and angiotensin II. Similarly, Calò and colleagues [80] have demonstrated that in macrophages Aldo induces the transcription of profibrotic genes such as the plasminogen activator inhibitor-1 (PAI-1). PAI-1 is a serine protease inhibitor, secreted by endothelial cells, VSMCs, platelets, hepatocytes, and adipocytes [81], which acts as a regulator of the fibrinolytic system. Importantly, Skurk and colleagues have demonstrated that obese people, in addition to elevated Aldo levels, have higher levels of PAI-1 [82] that may contribute to enhanced cardiac fibrosis and collagen secretion. All these data strongly support the notion that Aldo/MR system activation is a major culprit of cardiac fibrosis, an evidence supported by a myriad of *in vitro* and *in vivo* studies. Accordingly, Stockand and colleagues [7] have demonstrated that Aldo promotes the proliferation of myofibroblasts via activation of MAPK pathways contributing to the collagen deposition within the myocardium [8]. Similarly, Fraccarollo and coworkers [83] have shown that cardiac-specific deletion of the MR halts reactive fibrosis in response to myocardial ischemia, thus improving the reparative fibrotic response. Furthermore, in this study, the authors demonstrated that the loss of MR in cardiomyocytes leads to a reduced synthesis and release of proinflammatory cytokines and profibrotic factors. At the molecular level, it has been demonstrated that Aldo in fibroblasts increases the expression of TGF-*β*, as well as of CTGF, via MR activation, thus enhancing their function and increasing the production of matrix proteins in the myocardium [69]. Consonant to these findings, we have recently shown that downstream of the Aldo/MR signaling pathway, the activation of GPCR kinase GRK2 prompts a marked increase in CTGF expression in cardiomyocytes with a consequent augmented collagen deposition within the myocardium [54]. Interestingly, as per our study, GRK2 activation and subsequent CTGF expression are strictly dependent on AT1R activation [54]. Thus, this evidence validates the conclusions drawn in previous reports suggesting that AT1R signaling pathway activation is a major culprit for Aldo adverse effects in both the myocardium and vasculature [84–86]. In this regard, Robert and colleagues [87] have demonstrated that AT1R blockade is as powerful as MR antagonism in preventing cardiac fibrosis due to Aldo treatment. Similarly, Takeda and colleagues [88] showed that chronic infusion of Aldo robustly increases calcineurin expression and activity in the heart of uninephrectomized rats with a consequent augmented cardiac hypertrophic and fibrotic response. Of note, these effects of Aldo were significantly attenuated when rats were treated with a calcineurin inhibitor or with the AT1R-blocker, Losartan [88].

Taken together, these data support a potential role for Aldo mediating detrimental effects in the heart by interactions with the MR. Indeed, hyperaldosteronism is always associated to HF development. In particular, plasma aldosterone levels may reach almost twenty times the normal limits in patients with HF [89–91]. For this reason, Aldo/MR inhibition/antagonism is an attractive therapeutic strategy, as we shall see in the next section.

## 6. Targeting Aldosterone/Mineralocorticoid Receptor Signaling Pathway in HF Patients: From Bench to Bedside

As we learned above, Aldo is a major culprit in the physiopathology of cardiovascular disease [69, 92]. Importantly, several preclinical studies have shown that a link exists between Aldo or MR activation and cardiovascular risk factors. Most importantly, the presence of this association has been confirmed in human settings since MR antagonist addiction contributes to a reduction in mortality and HF hospitalization (ESC HF guidelines 2016 https://www.escardio.org/Guidelines/Clinical-Practice-Guidelines/Acute-and-Chronic-Heart-Failure): a direct correlation between Aldo and mortality in patients with severe HF was shown in the Cooperative North Scandinavian Enalapril Survival Study (CONSENSUS); the 6-month mortality rate was higher in HF patients with elevated Aldo levels [90, 92]. Notably, none of the patients enrolled in this trial was treated with an Aldo antagonist. Yet, in the last three decades, a number of *in vitro* and *in vivo* studies have demonstrated the salutary effects exerted by inhibition of the Aldo/MR system in disparate models of cardiovascular disorders.

For this reason, the Randomized Aldactone Evaluation Study (RALES) was generated to study the effects of the Aldo antagonist spironolactone in patients with systolic HF and LVEF ≤35% [93]. Severe symptomatic patients (1663 subjects with NYHA class III or IV) were enrolled in the trial and randomized to receive spironolactone or placebo: a 30% reduction in mortality was obtained in the spironolactone group. However, a disadvantage of MRA therapy is hyperkalemia. Indeed, following publication of the RALES trial, a relatively large number of hospitalizations and deaths from hyperkalemia were registered [94]. At the same time, a variety of common Aldo antagonism side effects were observed (i.e., gynecomastia in men) [95].

In the Emphasis trial (https://www.nejm.org/doi/full/10.1056/nejmoa1009492), HF patients with LVEF ≤35% and mild symptoms (NYHA II) (a total of 2737 individuals) were randomized to eplerenone or placebo groups: a 37% reduction in the primary composite endpoint of death from cardiovascular causes or hospitalization for HF was evident in the group of patients treated with eplerenone. Interestingly, the majority of the population was receiving ACE-I/ARBS and beta-blocker background therapy, making the results more impressive than the RALES trial, in which only about 10% of patients were also treated with *β*AR blockers.

In the wake of the encouraging data obtained from clinical and several preclinical animal models attesting the benefits afforded by Aldo antagonism on postischemic cardiac fibrosis, hypertrophy, and oxidative stress, it was hypothesized that an Aldo antagonist in post-MI patients with systolic heart failure would ameliorate both the remodeling and the function. For this reason, a new trial was designed using eplerenone, a drug with a similar structure to spironolactone but with higher specificity for MR and less side effects: the Eplerenone Post-Acute Myocardial Infarction Heart Failure Efficacy and Survival Study (EPHESUS) [96]. The authors randomized about 6600 subjects with acute HF, comprising post-MI HF-patients with EF ≤40%, to receive either eplerenone or placebo [96]. A significant decrease in mortality with a relative risk reduction of about 15%, and a further reduction of cardiovascular mortality and/or hospitalization, was observed in the eplerenone-treated group. Interestingly, the beneficial effects associated with Aldo antagonism began at 3 months from randomization and continued throughout the entire study duration, suggesting protection against cardiovascular morbidity and mortality.

The setting of acutely decompensated HF was explored by the recent small ATHENA-HF Randomized Clinical Trial (https://jamanetwork.com/journals/jamacardiology/article-abstract/2643429?resultClick=1). This study consisted of 360 randomized patients to assess the effect of high-dose spironolactone *vs* low dose or placebo on N-terminal pro-B-type natriuretic peptide (NT-proBNP) levels. No significant difference was detected in the primary end-point (log NT-proBNP reduction) or in the main secondary ones: all-cause mortality or HF hospitalization. Safety profile was also similar between the 2 groups with no significant changes in serum potassium and estimated glomerular filtration rate at 24, 48, 72, and 96 hours. Hence, this evidence that more studies concerning acute HF are needed and that findings obtained in one setting cannot be ipso facto extended to another one.

ESC guidelines for management of HF recommend spironolactone or eplerenone in all HFrEF patients with LVEF ≤35% who remain symptomatic after treatment with an ACEI and a β-blocker (ESC HF guidelines 2016).

Conversely, the role of MRA in HF with preserved left ventricular function (HFpEF), a common condition without established therapy, still remains unclear.

The Aldo-DHF trial (https://jamanetwork.com/journals/jama/fullarticle/1656252) was one of the first randomized trials on HFpEF population: the authors set the objective to determine whether or not spironolactone is superior to placebo in improving diastolic function and maximal exercise capacity, assessed with changes in diastolic function (*E*/*e*′) echocardiography and maximal exercise capacity (peak VO2) on cardiopulmonary exercise testing, respectively. NYHA II or III patients with HFpEF (422 subjects with EF ≥50% and evidence of diastolic dysfunction) were randomly assigned to a group receiving 25 mg of spironolactone once daily (*n* = 213) or to a group treated with placebo (*n* = 209). After 12 months of follow-up, MRA led to improved left ventricular diastolic function but no difference in maximal exercise capacity, patient symptoms, or quality of life was evident.

Similarly, the Treatment of Preserved Cardiac Function Heart Failure with an Aldo Antagonist (TOPCAT http://www.acc.org/latest-in-cardiology/clinical-trials/2014/06/04/12/40/topcat) trial enrolled 3445 patients with HFpEF (NYHA II or III and EF ≥45%), randomly assigned to receive spironolactone or placebo, to test differences in a primary composite outcome of composite cardiovascular mortality, cardiac arrest, and HF hospitalization [97–100]. Although the treated group did not benefit in terms of cardiovascular mortality and hospitalization for all causes, spironolactone has shown to significantly reduce the hospitalizations for decompensation and improve the quality of life assessed by the KCCQ (Kansas City Cardiomyopathy Questionnaire), especially in the lower FE subgroups [97–100].

Finally, the ALBATROSS Randomized Clinical Trial (https://www.ncbi.nlm.nih.gov/pubmed/27102506) tested the benefit of early MRA regimen in postmyocardial infarction, irrespective of the presence of HF or left ventricular (LV) dysfunction on primary composite outcome of death, resuscitated cardiac arrest, significant ventricular arrhythmia, indication for implantable defibrillator, or new or worsening HF at the 6-month follow-up. 1603 patients received, in 1:1 ratio, single intravenous bolus of potassium canrenoate followed by spironolactone (25 mg once daily, orally) in addition to standard therapy or standard therapy alone. The early addition of MRA to standard therapy did not show benefit in patients admitted for MI.

## 7. Conclusions

**Figure 2:**
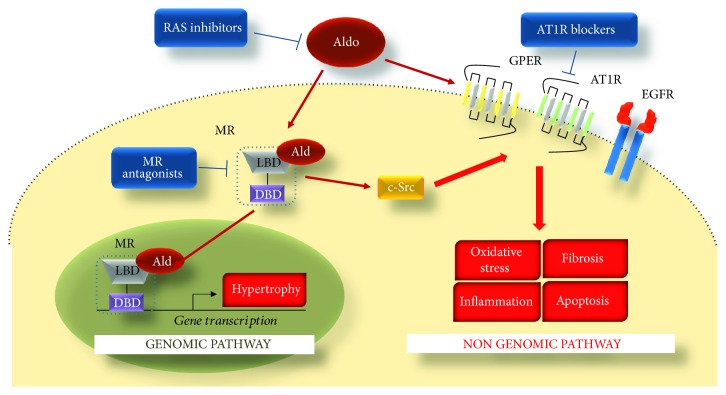
Aldo-mediated effects in cardiomyocytes. Aldo binds to MR (ligand-binding domain (LBD)) activating the following: a nongenomic pathway, which is mainly related to the cross-talk with other receptors, via c-Src, including the angiotensin II type 1 receptor (AT1R), the estrogen receptor (GPER), and the epidermal growth factor receptor (EGFR)), and a genomic pathway which is related to the translocation of the MR into the nucleus and the binding to the DNA (via a DNA-binding domain (DBD)). Highlighted are the cellular responses mediated by Aldo: hypertrophy (genomic pathway) and inflammation, fibrosis, hypertrophy, cell death, and oxidative stress (nongenomic pathway). These noxious effects can be blocked via renin-angiotensin system (RAS) inhibitors or via MR antagonists.

It is well consolidated that the sympathetic nervous system (SNS) and RAS hyperactivation are primary pathogenic drivers of HF [101–103]. Indeed, the augmented catecholamine (norepinephrine and epinephrine) levels chronically stimulate and dysregulate cardiac βARs [103–105], while exerting direct cardiotoxicity [105]. Chronic AT_1_R activation, mediated by Ang II, and the consequent increase in Aldo levels, leads to chronic MR activation and consequent cardiac dysfunction [54, 104]. As discussed in this review, Aldo maintains sodium homeostasis through direct action on the sodium excretion at the level of the distal tubules [91]. However, an excessive production and/or release of Aldo induces several “nonspecific” genomic and nongenomic effects (Figure 2) that, as widely demonstrated *in vitro* and *in vivo*, are conducive to vascular dysfunction and cardiac adverse remodeling. For this main reason, during the course of the last two decades, efforts have been made to efficiently block this noxious signalling via specific molecules that either block the RAS system (e.g., ACE inhibitors and Angiotensin II receptor blockers) or directly inhibit MR, as described above. In this context, it is worth noting that these agents are now considered a gold-standard HF patient care and are used in synergy with other mainstay therapies such as β-blockers [10, 54, 101, 104]. Ironically, despite that the survival of HF patients has been significantly improved, the prevalence of HF continues to increase. For this very reason, significant improvements in the prognosis and analysis of specific factors that contribute to HF development and progression must be made in order to further advance its therapeutic treatment.
